# Navigating Complexity: A Case Study on Hemophagocytic Lymphohistiocytosis Diagnosis and Management Challenges

**DOI:** 10.7759/cureus.59628

**Published:** 2024-05-04

**Authors:** Julie Nguyen, Nattapron Tun, Nick Burley, David Bolos

**Affiliations:** 1 Internal Medicine, St. Joseph's Medical Center, Stockton, USA; 2 Hematology and Oncology, Olive View-University of California Los Angeles Medical Center, Los Angeles, USA

**Keywords:** q fever, bone marrow biopsy, etoposide, epstein-barr virus, rickettsia, thrombocytopenia, non-malignant hematology, hemophagocytic lymphohistiocytosis (hlh), hemophagocyte

## Abstract

Hemophagocytic lymphohistiocytosis (HLH) is a severe inflammatory disorder that affects multiple organ systems and carries a high risk of mortality if untreated. Treatment typically involves immune suppression with steroids and cytotoxic drugs. This case report details the evaluation and management of an adult female presenting with atypical symptoms, aims to improve awareness and understanding of HLH in adults, and emphasizes the urgency of timely diagnosis and intervention.

## Introduction

Hemophagocytic lymphohistiocytosis (HLH) is a life-threatening inflammatory condition characterized by an aberrant and overwhelming immune response, impacting multiple organ systems and posing a substantial risk of mortality if left untreated. A significant subset of patients harbors inherent abnormalities impairing the regulation of immune responses mediated by activated macrophages and lymphocytes [1]. The diagnostic landscape of HLH remains challenging, primarily due to its diverse clinical presentations and a myriad of potential triggering factors. Consequently, accurately determining the prevalence and incidence of HLH proves challenging. One comparative study approximated the incidence to be one in 2,000 intensive care admissions [2]. Classically, patients may manifest with fever, splenomegaly, rash, lymphadenopathy, and neurological symptoms, although variations in presentation are common and may confound timely diagnosis [3]. Secondary HLH in adults poses an even more intricate diagnostic puzzle, often obscured by underlying triggers such as sepsis, uncontrolled malignancies, or autoimmune flares [1]. In order to be diagnosed with HLH, a patient must meet five of the eight diagnostic parameters: fever, splenomegaly, two or more cytopenias, hyperferritinemia, hypertriglyceridemia or hypofibrinogenemia, low or absent natural killer (NK) cell activity, elevated soluble interleukin 2 (IL-2) receptor alpha, or hemophagocytosis on bone marrow, lymph node biopsy, or cerebrospinal fluid [4]. Therapeutically, the cornerstone of HLH management revolves around immunosuppressive interventions complemented by cytotoxic chemotherapy [5]. Obtaining accurate estimates of adult fatality rates is challenging as it may be unclear whether death results from HLH or the underlying trigger. The absence of immunosuppressive and cytotoxic interventions significantly heightens mortality rates, emphasizing the urgency in prompt diagnosis and treatment initiation [1-2]. This case report analyzes the evaluation and management of a 69-year-old female presenting with atypical symptoms, shedding light on the intricate diagnostic nuances of HLH within a backdrop of overlapping differential diagnoses. The report underscores the complexities inherent in diagnosing HLH, aiming to contribute to the understanding and timely recognition of this life-threatening condition in adult populations.

## Case presentation

A 69-year-old Caucasian woman with a medical history of hypertension and subclinical hypothyroidism initially sought urgent care due to subjective fevers, cough, dyspnea, and reduced appetite. Initially diagnosed with a viral upper respiratory infection, she returned to urgent care three days later with metallic taste, delayed thinking, slurred speech, and hypoxia, prompting an emergency department referral. Vital signs revealed tachycardia and tachypnea, while the physical exam noted lethargy, limited orientation, dry mucous membranes, coarse breath sounds, and cold, cyanotic feet. Laboratory assessments were significant for a platelet count of 10 K/cumm, creatinine 1.19 mg/dL, total bilirubin 1.7 mg/dL, aspartate transaminase (AST) 234 U/L, alanine transaminase (ALT) 97 U/L, alkaline phosphatase 196 U/L, and lactate 3 mmol/L as seen in Table 1. Of note, her previous laboratory values about one month ago were all within normal limits. She was admitted to the hospital for severe sepsis of unknown origin. Imaging revealed lung tree-in-bud nodularity and periportal edema in the liver. Abdominal ultrasound showed no organ enlargement.

**Table 1:** The patient's notable laboratory values on the day of admission.

Laboratory test	Result	Normal value
Platelet count (K/cumm)	10	150-450
International normalized ratio	1.6	<1.1
Prothrombin (seconds)	14	11-13.5
Activated partial thromboplastin time (seconds)	35	19-30
Fibrinogen (mg/dL)	124	200-393
Haptoglobin (mg/dL)	35	40-200
Creatinine (mg/dL)	1.19	0.6-1.1
Total bilirubin (mg/dL)	1.7	0.1-1.2
Aspartate transaminase (U/L)	234	8-33
Alanine transaminase (U/L)	97	4-36
Lactate (mmol/L)	3	<2
Lactate dehydrogenase (U/L)	400	140-280

Hematology consultation was sought due to acute thrombocytopenia, raising concerns of thrombotic thrombocytopenic purpura (TTP), disseminated intravascular coagulation (DIC), malignancy, HLH, or immune thrombocytopenic purpura (ITP). Further evaluation indicated elevated prothrombin time, activated partial thromboplastin time (aPTT), international normalized ratio (INR), lactate dehydrogenase, low fibrinogen, and low haptoglobin (as seen in Table 1), suggesting DIC in the setting of severe sepsis. An ADAMTS13 measurement was ordered to rule out inherited TTP, but the outcome would not be available for another three days. Due to the absence of positive pan cultures and the patient's lack of clinical improvement, Infectious Disease specialists assessed for atypical infections including Q fever, *Rickettsia*, and Epstein-Barr virus (EBV) given her recent exposure to cats, birds, and rodents.

**Figure 1:**
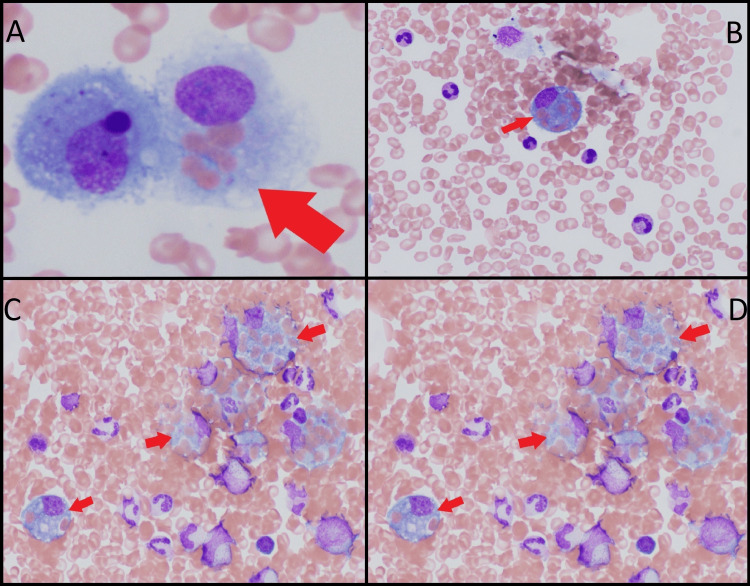
Hemophagocytosis seen on bone marrow biopsy. (A) Intact erythrocytes are within the cytoplasm of a single phagocytic histiocyte as indicated by the red arrow. (B-D) The presence of multiple nucleated cells within the cytoplasm of a single hematopoietic progenitor cell.

Despite interventions including platelet transfusion and cryoprecipitate administration, her condition deteriorated, leading to a transfer to the intensive care unit. High-risk suspicion for TTP warranted consideration for plasma exchange. However, the presence of elevated ferritin and fasting triglyceride levels supported the possibility of HLH, and a subsequently calculated HScore indicated a moderate to high probability. Because resource constraints did not allow for plasma exchange to be urgently started, intravenous dexamethasone was initiated as per the HLH-94 protocol. This initiation yielded transient improvement, and the diagnosis for HLH was further validated by subsequent bone marrow biopsy revealing hemophagocytosis as seen in Figure 1. Her soluble interleukin-2 receptor alpha was found to be greater than two standard deviations above age-adjusted laboratory-specific norms.

**Figure 2:**
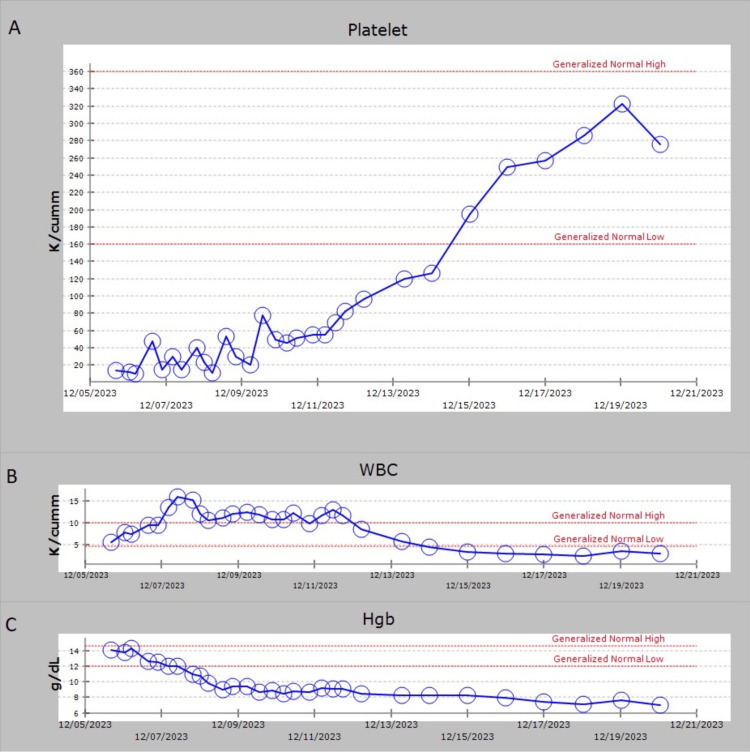
The y-axis illustrates the changes in (A) platelet count, (B) WBC count, and (C) Hgb over the duration from admission to discharge, while the x-axis denotes the timeline. Dexamethasone was started on 12/09/2023, followed by the initiation of etoposide on 12/11/2023. WBC: white blood cell count; Hgb: hemoglobin

**Figure 3:**
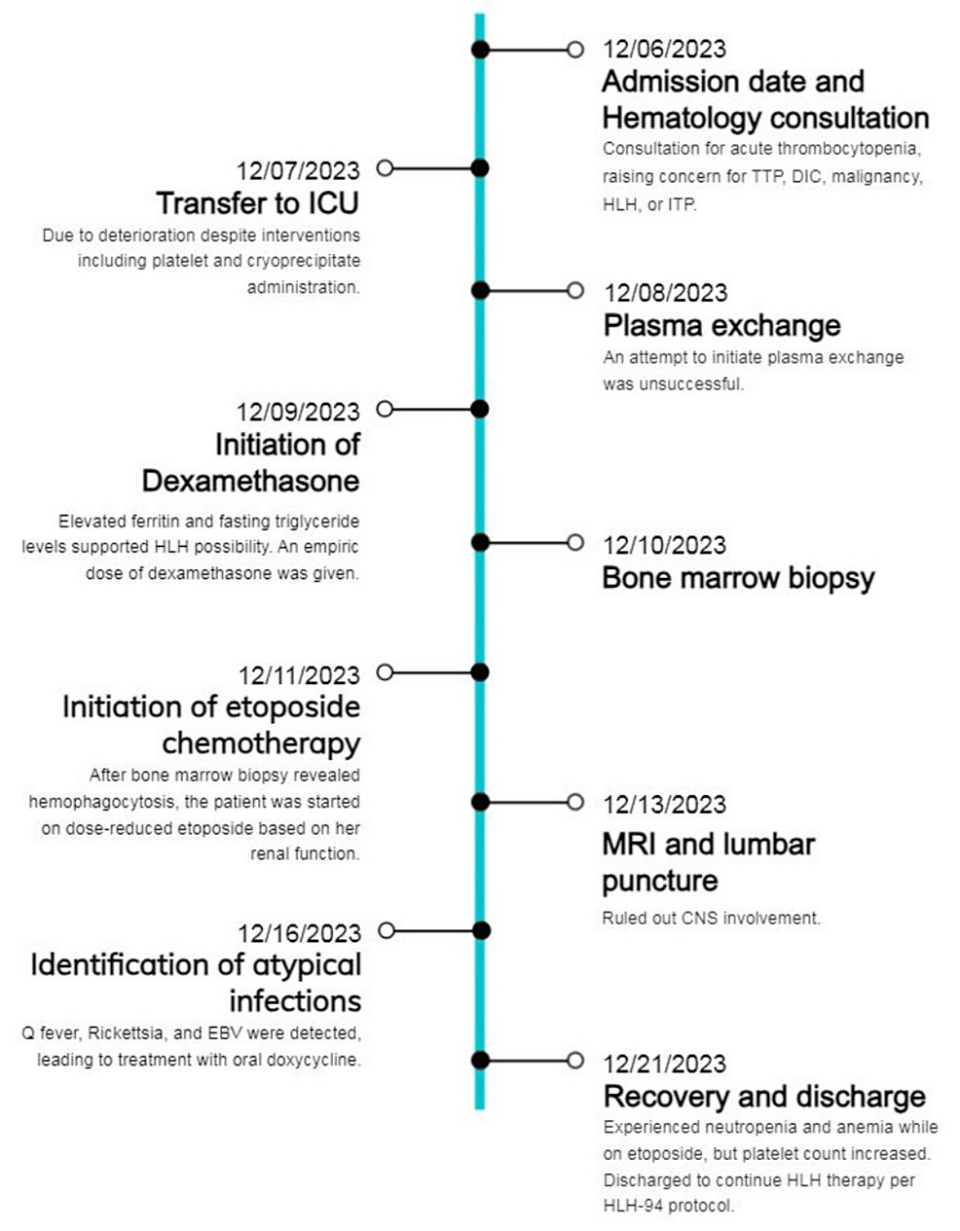
A timeline summarizing the events of the patient's hospital course. TTP: thrombotic thrombocytopenic purpura; DIC: disseminated intravascular coagulation; HLH: hemophagocytic lymphohistiocytosis; ITP: immune thrombocytopenic purpura; ICU: intensive care unit; CNS: central nervous system; EBV: Epstein-Barr virus

Despite an initial response, a decline in creatinine clearance to less than 50 mL/min necessitated dose-reduced etoposide chemotherapy at 75 mg/m^2^ as per the HLH-94 protocol, demonstrating significant therapeutic efficacy. Once her creatinine clearance improved, she was given the full dose of etoposide at 150 mg/m^2^. ADAMTS13 was found to be 56%. Further investigations with magnetic resonance imaging (MRI) of the brain with and without contrast and lumbar puncture ruled out central nervous system (CNS) involvement. She was later identified with Q fever immunoglobulin G (IgG) phase 2 positive 1:16, *Rickettsia typhi* immunoglobulin M (IgM) 1:64, and EBV IgG >3,000 as seen in Table 2. She received treatment with oral doxycycline 100 mg twice daily for 14 days. While on etoposide, she experienced neutropenia and anemia, but her platelet count increased to 338 K/cumm as seen in Figure 2A-2C. She was discharged to continue HLH therapy per HLH-94 protocol. Figure 3 presents a timeline summarizing the events during the patient's admission.

**Table 2:** Positive Q fever IgG, Rickettsia typhi IgM, and EBV IgG serologies. Ig: immunoglobulin; EBV: Epstein-Barr virus

Test	Result
Q fever IgG	Detected
Q fever IgG titer	1:16
Q fever IgM	Not detected
*Rickettsia typhi* IgG	Not detected
*Rickettsia typhi* IgM	Detected
*Rickettsia typhi* IgM titer	1:64
EBV IgG	Detected
EBV IgG titer	>3,000
EBV IgM	Not detected

## Discussion

The presented case underscores the intricate diagnostic challenges encountered in identifying HLH amidst a myriad of differential diagnoses, wherein atypical presentations and overlapping features often complicate timely recognition. The initial clinical picture, marked by subjective fevers, respiratory symptoms, encephalopathy, and acute, severe thrombocytopenia, invoked a broad array of differentials including TTP, DIC, malignancy, and ITP.

Elevated lactate dehydrogenase, low fibrinogen, and altered coagulation parameters within the context of severe sepsis initially suggested DIC. A unit of platelets and cryoprecipitate were transfused, but unfortunately her platelet count and fibrinogen did not improve. Because TTP is a life-threatening condition and her clinical status was deteriorating as evidenced by multi-organ failure, urgent plasma exchange was considered [6]. Unfortunately, because of a lack of available nursing staff, plasma exchange could not be initiated for several days. Subsequently, the patient was unresponsive to conventional measures including platelet and cryoprecipitate transfusions. This triggered a clinical pivot towards considering HLH, guided by exceptionally elevated ferritin and fasting triglyceride levels, characteristic of the syndrome [7]. The HScore, evaluating HLH probability, encompassed various clinical and laboratory parameters: temperature, organomegaly, cytopenia, ferritin, triglycerides, fibrinogen, AST levels, and bone marrow findings [8]. A score greater than 169 would be suggestive of a positive diagnosis. The patient's HScore was 196 before the bone marrow biopsy, indicating an 80-88% likelihood of HLH. Employing this algorithm assisted in navigating the diagnostic complexities of such cases.

The diagnostic criteria for HLH were initially proposed in 1991 using a standardized set of five diagnostic criteria for a prospective clinical trial. Later, in 2004, these criteria were revised for the HLH-2004 diagnostic criteria, encompassing eight diagnostic parameters [4]. To be diagnosed with HLH according to the HLH-2004 criteria, a patient must exhibit either an HLH-associated heterozygous genetic mutation or a minimum of five out of eight defined features. These features include fever exceeding 38.5°C, splenomegaly, two or more peripheral cytopenias, fasting triglycerides over 265 mg/dL, and/or hypofibrinogenemia (below 150 mg/dL), biopsy-confirmed hemophagocytosis, ferritin levels above 500 µg/mL, soluble IL-2 receptor alpha elevation by two standard deviations compared to age-adjusted laboratory norms, or reduced or absent NK cell activity [9]. Hemophagocytosis signifies a macrophage engulfing bone marrow cells [1]. Within 48 hours of admission, the patient fulfilled four of the criteria with fever, two peripheral cytopenias (anemia and thrombocytopenia), hypertriglyceridemia and hypofibrinogenemia, and hyperferritinemia. The challenge was the nonspecific nature of most of these features. In the absence of bone marrow biopsy confirming hemophagocytosis, severe infection could also elevate many of these parameters, rendering diagnosis intricate.

The administration of dexamethasone, following which transient improvement was observed, further validated the suspicion of HLH [4]. A subsequent bone marrow biopsy revealing hemophagocytosis confirmed the diagnosis. Following the HLH-94 protocol, we used decreasing doses of dexamethasone and etoposide over eight weeks [5]. A study using mice infected with lymphocytic choriomeningitis virus showed that etoposide significantly improved all symptoms of murine HLH and extended survival [10]. There was an impressive response to etoposide chemotherapy as seen in the improvement in platelet count as seen in Figure 2, emphasizing the pivotal role of cytotoxic agents in mitigating the aberrant immune activation characteristic of HLH.

This case had additional layers of complexity. Thorough investigation into the cause of the patient's systemic inflammatory response led to Infectious Disease specialists testing for several bacteria and viral infections on admission. The patient tested positive for Q fever, *Rickettsia typhi*, and EBV serologies, indicating that one or more of these infectious agents was likely responsible for the systemic inflammatory response. There have been reported cases of Q fever, *Rickettsia*, and EBV as causes of HLH [11-13]. The absence of CNS involvement, confirmed by neuroimaging and cerebrospinal fluid analysis as reported in CNS-HLH, elucidates the comprehensive diagnostic evaluation essential in excluding multi-organ involvement, a critical facet in HLH management [14].

The management of the patient within a community hospital setting presented distinctive challenges, primarily attributed to resource limitations that significantly impacted the decision-making process. The scarcity of resources posed considerable hurdles in promptly initiating interventions such as plasma exchange, crucial in suspected TTP. The lack of immediate availability of plasma exchange necessitated a meticulous reevaluation of the therapeutic strategy and ultimately the decision to initiate steroids. This deliberation underscored the intricate balance between various treatment modalities, weighing the urgency of interventions against resource availability and patient-specific factors to devise an optimal management plan.

## Conclusions

The case highlighted the complexity of diagnosing HLH amidst overlapping conditions. Initial evaluation pointed to DIC, but refractory responses led to suspect HLH, further validated by biopsy and response to dexamethasone. Concurrent infections added complexity to the diagnosis. Adherence to the HLH-94 protocol highlighted the importance of standardized treatment, offering a structured approach to managing HLH even beyond acute care. Altogether, this case serves as a poignant reminder of the intricate nature of HLH diagnosis, the multifaceted challenges in untangling overlapping differential diagnoses, and the criticality of prompt recognition and targeted therapeutic interventions.
